# Relationship between bone turnover markers and diabetic kidney disease in patients with type 2 diabetes

**DOI:** 10.3389/fendo.2025.1646690

**Published:** 2025-09-30

**Authors:** Xueyan Men, Peipei Yue, Weiwei Hao, Lei Zhang, En Chen, Jing Liu

**Affiliations:** ^1^ Department of Rheumatology, The First Affiliated Hospital of Zhengzhou University, Zhengzhou, Henan, China; ^2^ Department of Clinical Laboratory Medicine, The First Affiliated Hospital, Hengyang Medical School, University of South China, Hengyang, Hunan, China

**Keywords:** diabetic kidney disease, bone turnover markers, β-cross-linked C-telopeptide, risk factors, type 2 diabetes

## Abstract

**Objective:**

Diabetic kidney disease (DKD) is one of the most serious complications of type 2 diabetes mellitus (T2DM), and bone metabolism disorders show a close linkage to DKD. Thus, this study aimed to explore the association between bone turnover markers (BTMs) and DKD.

**Methods:**

In present cross-sectional study, serum BTMs were detected in 1433 hospitalized patients with T2DM. Logistic regression analysis was used to investigate the associations between osteocalcin (N-MID), β-cross-linked C-telopeptide (β-CTX), total type I collagen N-terminal propeptide (PINP), and the risk of DKD.

**Results:**

The circulation N-MID, β-CTX, and PINP levels were significantly lower in the DKD group compared with the non-DKD group (all P < 0.05), especially in male and aged < 60 subgroups. Serum BTM levels showed a weak correlations with certain glucose metabolism parameters–such as glycated hemoglobin, fasting blood glucose, C peptide, and fasting insulin−as well as alkaline phosphatase (ALP) levels and low-density lipoprotein (all P < 0.001). A weak negative correlation was also observed with the duration of diabetes (all P < 0.0001). In addition, β-CTX levels showed a minimal positive correlation with eGFR (r = 0.057, P=0.036) and a modest correlation with ALP (r = 0.31, P < 0.0001). After adjusting for potential confounders, higher serum β-CTX levels were independently associated with a lower risk of DKD. However, no significant associations were found among serum N-MID, PINP, and the risk of DKD.

**Conclusion:**

BTM levels were significantly decreased in patients with DKD. Lower β-CTX levels were independently associated with a larger prevalence of DKD after adjusting for potential confounders, suggesting that serum β-CTX may be an independent marker associated with the risk of DKD.

## 1 Introduction

Diabetic kidney disease (DKD) is one of the most serious chronic microvascular complications of type 2 diabetes mellitus (T2DM), and bone metabolism disorders show a close link to the occurrence of DKD (1, 2). The presence of DKD not only increases the complexity of diabetes management but also considerably increases the risks of cardiovascular and all-cause mortality (3, 4). The detection of possible molecular markers related to DKD is essential for screening populations with a high risk of DKD (5).

Increasing evidence has revealed that bone-derived factors and hormones serve as important regulators of energy metabolism (6). The prevalence of T2DM and bone metabolism disorders increase with age (7). Chronic hyperglycemia reduces osteoblast numbers, impairs bone formation, decreases bone mineralization, and enhances osteoclast activity, which result in an imbalance in bone metabolism, particularly in patients with T2DM (8, 9). However, the association between bone turnover markers (BTMs) and DKD remains unclear.

Bone turnover refers to a dynamic process that includes the formation of new bone and the resorption of old bone. This process generates many BTMs from bone cells, which provide a noninvasive marker for the evaluation of bone metabolism status (10, 11). BTMs comprise bone formation and resorption markers, such as N-terminal osteocalcin (N-MID), total type I collagen N-terminal propeptide (PINP), and β-cross-linked C-telopeptide of type I collagen (β-CTX). In addition to the use of BTMs as indicators of bone formation and resorption, BTMs are related to glucose and lipid metabolism (12–14).

A previous study demonstrated that adverse metabolic conditions related to lower serum osteocalcin levels, such as obesity, insulin resistance, and hyperglycemia, may further deteriorate and contribute to the development of DKD, which indicates that the serum BTMs may be related to the risk of DKD (15). However, there no cohort-based studies have assessed BTMs as a predictor of incident DKD. Therefore, we aimed to explore the associations of serum BTM levels with the risk of DKD on the basis of a cross-sectional study.

## 2 Methods

### 2.1 Study populations

For this cross-sectional study, patients with T2DM aged ≥ 18 years were recruited from the First Affiliated Hospital of Zhengzhou University. T2DM was diagnosed in accordance with the criteria established by the American Diabetes Association (16). The exclusion criteria of study populations were as follows: (1) lack of serum BTMs data or medical history; (2) experience of acute complications of diabetes, infections, autoimmune diseases, or malignancies; (3) receipt of steroid or thyroid hormone treatment; (4) intake of drug that may influence serum BTM levels; (5) receipt of hemodialysis or peritoneal dialysis or estimated glomerular filtration rate (eGFR) less than 30 mL/min/1.73 m^2^. In total, 1433 participants were enrolled in the present analysis. This retrospective study was approval by the Institutional Review Broad of the First Affiliated Hospital of Zhengzhou University (2021-KY-0504).

### 2.2 Clinical parameters measurement

A questionnaire was used to collect data on participant demographics, including smoking and drinking status, medical history, and other clinical information. Systolic blood pressure (SBP), and diastolic blood pressure (DBP) were measured after at least 10min of rest. Body mass index (BMI) was calculated as body weight (kg) divided by the square of height (m^2^). Blood samples were collected after overnight fasting. Electrical chemiluminescent immunoassay was conducted to detect the fasting insulin and serum BTM levels, including N-MID, β-CTX, and PINP. Glycated hemoglobin (HbA1c) was measured through high-performance liquid chromatography. Fasting blood glucose (FBG), serum total cholesterol (TC), triglyceride (TG), high-density lipoprotein cholesterol (HDL-C), and low-density lipoprotein cholesterol (LDL-C) were assessed by an auto-biochemical analyzer.

### 2.3 Definition of DKD

Diabetes was diagnosed based on a history of diabetes, FBG ≥ 7.0 mmol/L, and/or HbA1c ≥ 6.5% according to the American Diabetes Association guidelines. The eGFR was calculated as the Chronic Kidney Disease Epidemiology Collaboration equation. Albuminuria was defined as a urinary albumin-to-creatinine ratio (UACR) ≥ 30 mg/g on at least two separate tests conducted 3 to 6 months apart. A reduced eGFR was described as <60 mL/min/1.73 m^2^ on at least two separate tests conducted 3 to 6 months apart. The clinical diagnosis of DKD based on the presence of albuminuria and/or decreased eGFR in the absence of other primary causes of kidney damage (17). Study populations were classified into DKD and non-DKD groups according to the presence of kidney damage.

### 2.4 Statistical analysis

Normality testing were conducted, continuous variables are expressed as median (interquartile range), whereas categorical variables expressed as proportions. Differences in anthropometric and biochemical variables between two groups were evaluated through the Mann–Whitney U test or χ^2^-test as appropriate. Spearman correlation analysis was conducted to estimate the relationships between serum BTMs and other clinical variables. Logistic regression models were constructed to assess the odds ratio (OR) and 95% confidence interval (CI) for incident DKD after the adjustment for potential confounders. All statistical analyses were performed using SPSS Statistics 26.0. P < 0.05 was considered statistically significant.

## 3 Results

### 3.1 Characteristics of study population

**Table 1** shows the baseline characteristics of the study participants. The final analysis included 1433 patients with T2DM. Among the populations, 1012 (70.6%) were diagnosed without DKD (non-DKD), and 421 (29.4%) were diagnosed with DKD. Compared with non-DKD group, patients with DKD were older and had significantly higher percent of male participants (71.3%), BMI, duration of diabetes (DD), and levels of FBG, HbA1c, TG, alkaline phosphatase (ALP), serum creatinine, UACR, and SBP (all P< 0.05). No differences were observed in TC, LDL, C peptide, and fasting insulin levels between the two groups. However, HDL, albumin (ALB), and eGFR were significantly lower in the patients with DKD compared with those without DKD (all P< 0.05).

**Table 1:** Baseline characteristics of the participants by DKD.

	Non-DKD (n=1012)	DKD (n=421)	P value
Age, years	50 (41, 57)	54 (45, 62)	0.0001
Male, n (%)	643 (63.5%)	300 (71.3%)	0.005
Hypertension, n (%)	374 (37.0%)	234 (55.6%)	<0.001
CVD, n (%)	88 (8.7%)	43 (10.2%)	0.364
Smoking, n (%)	249 (24.6%)	106 (25.2%)	0.819
Drinking, n (%)	219 (21.6%)	85 (20.2%)	0.54
DD, years	3 (0.3, 8.0)	6 (1.0, 14.8)	<0.001
BMI, kg/m^2^	25.4 (23.0, 28.1)	25.9 (23.5, 28.3)	0.003
HbA1c, %	8.2 (6.9, 10.1)	9.0 (7.3, 10.5)	<0.001
FBG, mmol/L	7.4 (5.9, 9.8)	7.5 (6.2, 10.6)	0.039
Serum Cr, μmol/L	62 (53.0, 72.0)	66 (56.0, 81.8)	<0.001
UA, μmol/L	293.5 (240.8, 354.8)	315.5 (256.3, 375.0)	0.0001
eGFR, ml/min/1.73 m^2^	107.7 (99.9, 116.1)	102.0 (88.6, 113.7)	<0.001
ALP, U/L	71 (60.0, 85.0)	72 (61, 85.0)	0.018
ALB, g/L	44.2 (41.4, 47.0)	43.6 (40.4, 46.1)	<0.001
TC, mmol/L	4.5 (3.8, 5.2)	4.3 (3.5, 5.0)	0.99
TG, mmol/L	1.7 (1.1, 2.4)	1.8 (1.2, 2.6)	0.001
HDL, mmol/L	1.06 (0.87, 1.24)	1.0 (0.85, 1.24)	0.0009
LDL, mmol/L	2.7 (2.1, 3.2)	2.5 (1.8, 3.2)	0.47
UACR, mg/g	4.4 (2.6, 8.2)	59.9 (27.6, 218.0)	<0.001
C peptide, ng/mL	1.96 (1.26, 2.87)	1.84 (1.25, 2.64)	0.926
Insulin, μU/mL	5.2 (2.7, 9.4)	5.2 (2.4, 9.5)	0.823
SBP, mmHg	132 (122, 140)	135 (125, 149)	<0.001
DBP, mmHg	83 (75, 89)	84 (77, 89)	0.0099

### 3.2 Serum BTM levels in different age and sex subgroups

In all study populations, compared with non-DKD group, those with DKD showed significantly lower levels of N-MID, β-CTX, and PINP (all P < 0.05). Considering the trend of serum BTM levels based on age and sex, we further divided the study populations into four groups according to sex and age. In the male subgroup, serum N-MID, β-CTX, and PINP levels were lower in the DKD than in the non-DKD groups (P < 0.05). Furthermore, in the aged < 60 subgroup, significant differences were observed in the BTMs between DKD and non-DKD patients (all P < 0.005). Interestingly, in female and aged ≥ 60 subgroups, no differences were found in the serum BTM levels between above two groups (**Table 2**).

**Table 2:** The levels of BTMs in patients with T2DM according to age and sex.

Variables		Non-DKD	DKD	P value
All	N-MID	12.3 (10.2, 15.7)	12.2 (9.2, 15.5)	0.006
	β-CTX	0.39 (0.27, 0.56)	0.35 (0.25, 0.47)	0.002
	PINP	36.4 (28.1, 48.9)	33.8 (27.9, 44.7)	0.024
Male	N-MID	12.1 (10.2, 15.0)	11.2 (9.1, 14.1)	<0.001
	β-CTX	0.38 (0.28, 0.55)	0.34 (0.24, 0.47)	<0.001
	PINP	35.2 (26.8, 45.2)	32.5 (25.3, 42.2)	0.022
Female	N-MID	13.0 (10.2, 17.2)	13.1 (10.2, 17.5)	0.479
	β-CTX	0.38 (0.28, 0.55)	0.42 (0.27, 0.53)	0.925
	PINP	38.5 (29.3, 53.6)	38.5 (28.7, 53.5)	0.991
Age≥60	N-MID	12.7 (10.2, 16.2)	12.6 (9.7, 16.7)	0.474
	β-CTX	0.37 (0.27, 0.56)	0.36 (0.23,0.51)	0.260
	PINP	37.1 (27.1, 48.3)	36.4 (26.5, 50.7)	0.929
Age<60	N-MID	12.3 (10.1, 15.4)	11.5 (9.2, 14.2)	0.002
	β-CTX	0.39 (0.28, 0.55)	0.35 (0.24, 0.49)	0.004
	PINP	36.6 (27.9, 47.7)	33.3 (26.0, 42.9)	0.005

### 3.3 Relationships of serum BTM levels with other clinical parameters

Spearman correlation analyses were conducted to assess the relationship of serum BTM and other clinical variables (**Table 3**). A weak correlation was observed between serum BTM levels and some glucose metabolism parameters, including HbA1c, FBG, C peptide, and fasting insulin levels. Serum BTM levels were also weakly positively correlated with ALP levels (all P < 0.0001) and LDL (all P < 0.001), and weakly negatively related with DD (all P < 0.0001). No meaningful liner correlation was detected between BTM levels and serum creatinine, TC, UACR, or SBP. Furthermore, serum β-CTX levels showed a slight negative relation with age (r = -0.077, P=0.003), a minimal positive correlation with eGFR (r = 0.057, P=0.036), and a moderate correlation with ALP (r = 0.31, P < 0.0001).

**Table 3:** Correlation between BTMs and other parameters in patients with T2DM.

Variables	N-MID		β-CTX		PINP	
	r	P value	r	P value	r	P value
Age	0.050	0.059	-.077	0.003	-0.021	0.431
DD	-.116	<0.001	-.223	<0.001	-.137	<0.001
BMI	-.078	0.003	-0.022	0.418	0.038	0.162
HbA1c	-.165	<0.001	-0.022	0.426	-.075	0.006
FBG	-.173	<0.001	-.074	0.006	-.129	<0.001
Serum Cr	0.032	0.238	-0.035	0.187	-0.038	0.153
UA	-0.038	0.160	-.070	0.009	-0.040	0.139
eGFR	-.107	<0.001	.057	0.036	-0.002	0.946
ALP	.280	<0.001	.310	<0.001	.359	<0.001
ALB	-0.022	0.412	-0.043	0.112	-.071	0.008
TC	0.030	0.267	0.044	0.101	0.015	0.567
TG	-.084	0.002	-0.049	0.070	-0.047	0.083
HDL	.081	0.003	0.018	0.510	-0.009	0.750
LDL	.093	<0.001	.087	0.001	.088	0.001
UACR	-.052	0.105	-0.022	0.493	-0.001	0.963
C peptide	.091	0.002	.087	0.004	.101	0.001
Insulin	.154	<0.001	.105	0.005	.202	<0.001
SBP	0.041	0.119	0.041	0.122	-0.013	0.622
DBP	0.027	0.307	.060	0.023	-0.017	0.525

### 3.4 Univariable analysis of correlation between clinical parameters and DKD

Univariable analysis revealed the positive association of older age, male gender, longer DD, higher BMI, poor glucose control (high FBG and HbA1c levels), elevated levels of serum creatinine, ALP, SBP, and UACR with a higher risk of DKD (all P < 0.05). The presence of hypertension at diagnosis was associated with a higher risk of DKD (OR=2.14; 95% CI=1.70–2.69; P < 0.0001). In addition, low eGFR, ALB, and serum β-CTX (OR=0.408; 95% CI=0.238–0.70; P=0.001) levels exhibit a great incidence of DKD (**Table 4**). However, no significant associations were found among serum N-MID, PINP levels, and the risk of DKD.

**Table 4:** Logistic regression analyses of the association between clinical parameters and DKD.

Variables	ORs	95% CI		P value
		Lowest	Highest	
Sex, (Male vs. Female)	1.423	1.112	1.821	0.005
Age, years	1.021	1.011	1.031	<0.001
DD, years	1.080	1.062	1.099	<0.001
Hypertension, Yes vs. No	2.14	1.70	2.69	<0.001
CVD, Yes vs. No	1.194	0.814	1.753	0.364
BMI, kg/m^2^	1.041	1.013	1.070	0.004
HbA1c, %	1.116	1.059	1.175	<0.001
FBG, mmol/L	1.035	1.003	1.068	0.033
Serum Cr, μmol/L	1.027	1.020	1.034	<0.001
eGFR, ml/min/1.73m^2^	0.972	0.965	0.978	<0.001
ALP, U/L	1.007	1.002	1.012	0.005
ALB, g/L	0.908	0.883	0.934	<0.001
TC, mmol/L	1.012	0.919	1.115	0.804
LDL, mmol/L	0.993	0.880	1.120	0.906
N-MID, ng/mL	0.991	0.969	1.013	0.425
β-CTX, ng/mL	0.408	0.238	0.700	0.001
PINP, ng/mL	0.995	0.988	1.001	0.090
UACR, mg/g	1.927	1.725	2.152	<0.001
UMALB, mg/L	1.064	1.052	1.075	<0.001
C peptide, ng/mL	1.038	0.963	1.118	0.328
Insulin, μU/mL	0.995	0.981	1.008	0.437
SBP, mmHg	1.021	1.014	1.029	<0.001
DBP, mmHg	1.016	1.006	1.028	0.003

### 3.5 Association of serum BTM levels with the risk of DKD

To explore the associations of serum BTMs with the risk of DKD, binary logistics regression analyses were conducted. Clinical and biochemical variables were adjusted for, including age, sex, drinking status, smoking status, hypertension, history of cardiovascular disease (CVD), BMI, DD, HbA1c, eGFR, ALP, SBP, and TG. The data demonstrate the significant association of serum β-CTX levels with DKD risk. Without any adjustments in Model 1, higher β-CTX levels were associated with a decreased incident of DKD (**Table 5**). After controlling for various confounders in Models 2, 3, and 4, the inverse associations between β-CTX and the risk of DKD remained statistically significant. In Model 4, adjustment for confounders revealed that low β-CTX levels were an independent risk factor for incidence of DKD (OR=0.38; 95% CI=0.19–0.75; P=0.006).

**Table 5:** Logistics regression analyses the risk factors of DKD.

Variables		ORs	95% CI		P value
Model 1	N-MID	0.991	0.969	1.013	0.425
	β-CTX	0.408	0.238	0.700	0.001
	PINP	0.995	0.988	1.001	0.090
Model 2	N-MID	0.996	0.973	1.019	0.712
	β-CTX	0.501	0.290	0.865	0.013
	PINP	0.997	0.991	1.003	0.372
Model 3	N-MID	0.996	0.973	1.019	0.732
	β-CTX	0.471	0.269	0.827	0.009
	PINP	0.997	0.990	1.003	0.293
Model 4	N-MID	1.002	0.974	1.032	0.868
	β-CTX	0.380	0.192	0.754	0.006
	PINP	0.998	0.990	1.005	0.543

Model 1 was unadjusted.

Model 2 was adjusted for age and sex.

Model 3 was adjusted for age, sex, hypertension, CVD history, drinking, smoking status, and BMI.

Model 4 was adjusted for adjusted for age, sex, hypertension, CVD history, drinking, smoking status, BMI, duration of diabetes, HbA1c, eGFR, ALP, SBP, and TG.

## 4 Discussion

DKD represents the most common diabetic chronic microvascular complication, and bone metabolism disorders are closely linked to its occurrence (18). The relationship between BTMs and DKD risk remains unclear. Present study revealed that the levels of serum N-MID, β-CTX, and PINP significantly decreased in the patients with DKD. Weak associations were found between serum BTM levels and glucose metabolism parameters, including FBG, C peptide, HbA1c, and fasting insulin levels, as well as positive correlations with ALP and LDL, and a negative correlation with DD. After the adjustment for potential confounders, higher serum β-CTX levels were independently related with a lower risk of DKD. These results indicate that lower β-CTX levels are associated with an increased risk of DKD.

Bone turnover rate varies based on individual variables, with age and sex serving as the most important variables that determine bone remodeling (19). Thus, considering the changes of serum BTM levels, we further divided the study populations into four groups according to age and sex. In the male subgroup, serum N-MID, β-CTX, and PINP levels were significantly lower in the DKD than non-DKD groups. Furthermore, in the age<60 subgroup, significant differences were founded in serum BTM levels between DKD and non-DKD patients. Interestingly, in female and age≥60 subgroups, the serum BTM levels showed no significant difference. Thus, consistent with previous observations, variables of bone turnover rate lead to age- and sex-specific differences in circulating BTM levels (20, 21). This variability in serum BTM levels may account for the inconsistent findings in previous studies on the relationships of circulating BTM levels with glucose homeostasis (13, 22). Notably, the difference of age and sex in bone turnover rates must be considered in the evaluation of the serum BTM levels in patients with T2DM.

Osteocalcin is the most abundant noncollagenous protein secreted by osteoblasts, and it plays crucial roles in various physiological processes, including bone metabolism and energy metabolism (10). The instability of intact osteocalcin results from the cleavage of its C-terminal sequence to produce a large N-terminal osteocalcin fragment (N-MID) (23). Osteocalcin has also been involved in the stimulation of islet beta cells to release insulin and thereby enhances insulin sensitivity (24). A cross-sectional study revealed a lower serum osteocalcin level in patients with diabetes; after follow-up for 4 years, the low serum osteocalcin group exhibited an increased risk of T2DM, impaired fasting glucose, and insulin resistance in general populations (25). These results demonstrate that serum osteocalcin may be involved in glucose metabolism and associated with the risk of diabetes. However, the underlying mechanisms should be explored in future prospective studies.

A previous research unveiled a significant positive correlation between N-MID and UACR in patients with T2DM, inconsistent with our findings (26). Another cross-sectional study revealed that T2DM patients with micro or macro-albuminuria had lower osteocalcin levels compared with patients without such a condition (27). A prospective study involving patients with T2DM concluded that low osteocalcin levels were relevant to an increased risk of incident DKD (25). However, no association was found between serum N-MID and DKD risk after the adjustment for confounders in the present study. The potential cause may be the different study populations and our cross-sectional research. Furthermore, N-MID from the cleavage of the C-terminal sequence of osteocalcin displayed better stability during storage than intact osteocalcin in serum (23).

The present study observed that weak correlation between serum BTM levels and several glucose metabolism parameters, such as FBG, C peptide, HbA1c, and fasting insulin levels. However, the correlation coefficients are small, suggesting a lack of strong linear relationship. We suspected that the relationship might be non-linear. A cross-sectional cohort study previously reported a significant association between higher osteocalcin levels and improved metabolic parameters, which appears partially consistent with the weak relationships observed here (28). Thus, BTMs may correlate positively with glycemic metabolism status, and lower serum BTM levels are associated with the incidence of T2DM (29). However, a prospective investigation should be implemented to assess the relationship of serum BTMs and the risk of T2DM.

β-CTX serves as a degradation product of type I collagen, and it reflects the biological activity of osteoclasts and the extent of bone resorption (30). β-CTX levels showed a slight negative correlation with age and minimal positive relation with eGFR, alongside a moderate correlation with ALP. Although very weak, a trend of increasing eGFR was observed with higher β-CTX levels. Previous research highlighted a positive correlation between β-CTX and eGFR in diabetic patients, which is consistent with the current findings (31). In addition, weak correlations were found between serum β-CTX levels and certain glucose metabolism parameters, which is tentatively consistent with some previous studies (31, 32). These outcomes suggest a potential—though weak—link between β-CTX and glucose metabolism. Nonetheless, the potential mechanism by which β-CTX affects glucose metabolism remains unclear and warrants further investigation.

To explore the independent associations of BTMs with the risk of DKD, binary logistics regression analyses were constructed. After adjusted for potential confounders, including clinical and biochemical variables, the results suggest that serum β-CTX levels are independently related to the risk of DKD. These findings highlight the utility of β-CTX as a biomarker for kidney disease, aiding in the identify patients at increased risk for DKD. This finding has evident clinical implications for the management of bone metabolism and kidney damage in T2DM patients. The observed association may be explained by the bone-kidney axis, where declining renal function disrupts mineral metabolism, potentially suppressing bone turnover and making low β-CTX a consequence rather than a cause of DKD (33). Future mechanistic studies are warranted to elucidate this potential crosstalk.

The present study included a large sample population, which included hospitalized patients with T2DM and ensured collect comprehensive data. This ensured us to adjust for common confounders. Thus, based on our findings, attention should be pay to the dynamic monitor serum β-CTX, keeping optimal serum β-CTX levels might be associated with a lower risk of DKD development. In clinical practice, measuring β-CTX could potentially be integrated into risk stratification algorithms to identify T2DM patients at highest risk for DKD who might benefit from more intensive monitoring or management.

Nevertheless, present study encountered some limitations. First, in this single-center based cross-sectional research, selection bias could not be avoided. Second, this study detected BTMs at a single time point and did not allow for explore the dynamic changes of serum BTMs and their association with the risk of DKD. Third, all study populations were from the central region of China, which may limit the generalizability of the findings to other region given that bone metabolism vary among different races, regions and clinical setting factors. Lastly, potential confounding factors may influencing serum BTMs levels, such as physical exercise, dietary habits, and sun exposure, were could not include in this analysis.

## 5 Conclusion

Serum BTM levels are significantly decreased in patients with DKD. Lower levels of β-CTX are associated with a higher prevalence of DKD. This suggests a potential link between decreased β-CTX and the risk of DKD. These results could help clinicians develop preventive and targeted treatment strategies for patients with T2DM.

## Data Availability

The raw data supporting the conclusions of this article will be made available by the authors, without undue reservation.
